# Is there a relationship between symptoms and types of endometriosis according to #ENZIAN? A comparative study of preoperative questionnaires

**DOI:** 10.1007/s00404-025-08072-w

**Published:** 2025-06-28

**Authors:** Elvin Piriyev, Clara Mennicken, Sven Schiermeier, Thomas Römer

**Affiliations:** 1 University Witten-Herdecke, Witten, Germany; 2 University of Cologne, Cologne, Germany; 3 Department of Obstetrics and Gynecology, Academic Hospital Cologne Weyertal University of Cologne, Weyertal 76, 50933 Cologne, Germany

**Keywords:** Endometriosis, Symptoms, #ENZIAN, Digestive symptoms, Questionnaires

## Abstract

**Objective:**

The primary objective was to evaluate the relationship between these three groups and digestive symptoms. The secondary objective was to evaluate all symptoms in all groups.

**Study design:**

It was a retrospective comparative analysis of preoperative questionnaires. Three groups of patients were compared: Group 1 Patients with only peritoneal endometriosis ± adnexal adhesions and adenomyosis (P ± T and FA), Group 2 Patients with DIE, excluding the digestive system, and/or cystic ± peritoneal and adnexal adhesions and adenomyosis (O, A, B ± P, T, and FA), Group 3 Patients with DIE of the digestive system (C, FI) ± other localizations.

**Results:**

This retrospective study of 233 preoperative questionnaires explored symptom profiles across #ENZIAN-classified endometriosis types. No overall symptom differences were found, but severe dyschezia (VAS ≥ 5) correlated with bowel involvement (C compartment), dyspareunia corelated with adenomyosis (FA compartment), and chronic pelvic pain was lower in bowel DIE (Group 3) than in peritoneal/ovarian groups. Symptom questionnaires may guide surgical referral despite imaging limitations.

**Conclusion:**

While these imaging modalities can help identify DIE and endometriomas, they are less effective in detecting superficial peritoneal lesions, which can also cause significant symptoms. For this reason, even though symptom questionnaires are not definitive diagnostic tools, they may serve as an important starting point for further investigation and referral for surgical evaluation.

## What does this study adds to the clinical work

**Table:** 

Symptom questionnaires can aid early identification of endometriosis but are not sufficient for definitive diagnosis. Combining symptom profiles with advanced imaging and surgical evaluation offers a more accurate and holistic approach.	

## Introduction

Endometriosis is one of the most common benign gynecological diseases, characterized by the presence of endometrium-like tissue and/or stroma outside the uterus, usually associated with an inflammatory process. The pathogenesis of endometriosis remains largely unknown [1, 2]. The epidemiology of endometriosis is poorly understood [3]. However, according to available data, up to 10% of women of reproductive age may have endometriosis. Asymptomatic women might have a prevalence of 2–11%. The prevalence of endometriosis among infertile women is 25–50% [4]. In women with pelvic pain, the prevalence of endometriosis can be as high as 21%. Among symptomatic adolescents, the prevalence of endometriosis ranges from 49% in those with chronic pelvic pain to 75% in those with pain unresponsive to medical treatment [5].

Endometriosis presents with diverse manifestations, ranging from superficial peritoneal and serosal lesions to ovarian endometriotic cysts (endometriomas) and nodules deeper than 5 mm (deep endometriosis), frequently accompanied by scarring (fibrosis) and adhesions [6]. The diagnosis of endometriosis can only be definitively confirmed through surgical visualization and histological examination, typically via laparoscopy. However, endometriomas and deep endometriosis may also be identified using imaging modalities such as ultrasonography or MRI [7].

Accurate classification of endometriosis is essential for understanding disease severity, identifying lesion locations, and evaluating clinicopathological outcomes. The #ENZIAN classification is among the most widely accepted systems, offering a comprehensive assessment of peritoneal, ovarian, and deep endometriosis across different compartments, as well as tubo-ovarian adhesions based on surgical observations [8, 9]. Additionally, the #ENZIAN classification can be applied using imaging modalities such as ultrasound and MRI [9]. In contrast to the revised American Society for Reproductive Medicine (rASRM) classification [4], which primarily addresses adhesions and endometriomas but lacks precision in describing the location and extent of deep endometriosis, the #ENZIAN system enables a more detailed evaluation of all endometriotic lesions. This facilitates effective communication among surgeons, diagnostic specialists, and reproductive health professionals.

Up to 70% of women with endometriosis are symptomatic [10, 11]. The primary clinical symptom of endometriosis is dysmenorrhea [10]. The most common symptoms are often referred to as the “4 Ds” of endometriosis (dysmenorrhea, dyspareunia, dysuria, dyschezia) [11]. However, symptoms are not limited to the “4 Ds”. The development of chronic pelvic pain (CPP), digestive symptoms including nausea, vomiting, diarrhea, obstipation, and bloating, as well as urinary symptoms such as pollakiuria and cystitis, can also occur [12–15]. Furthermore, in cases of DIE in the bowel or bladder, catamenial blood in stool or urine may occur [16, 17].

To the best of our knowledge, no studies have explored the correlation between the symptoms and endometriosis type based on #ENZIAN classification. The primary objective was to evaluate the relationship between these three groups and digestive symptoms. The secondary objective was to evaluate all symptoms in all groups.

## Method and materials

**Fig. 1:**
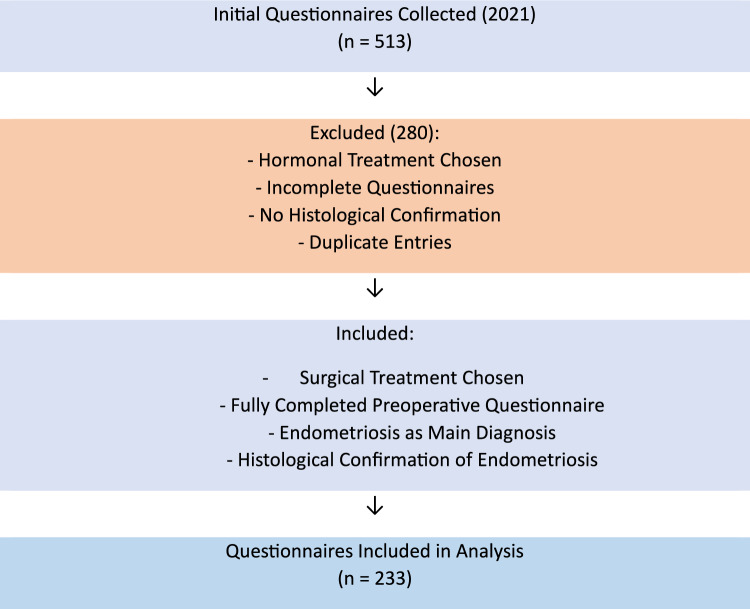
Questionnaire

**Fig. 2:**
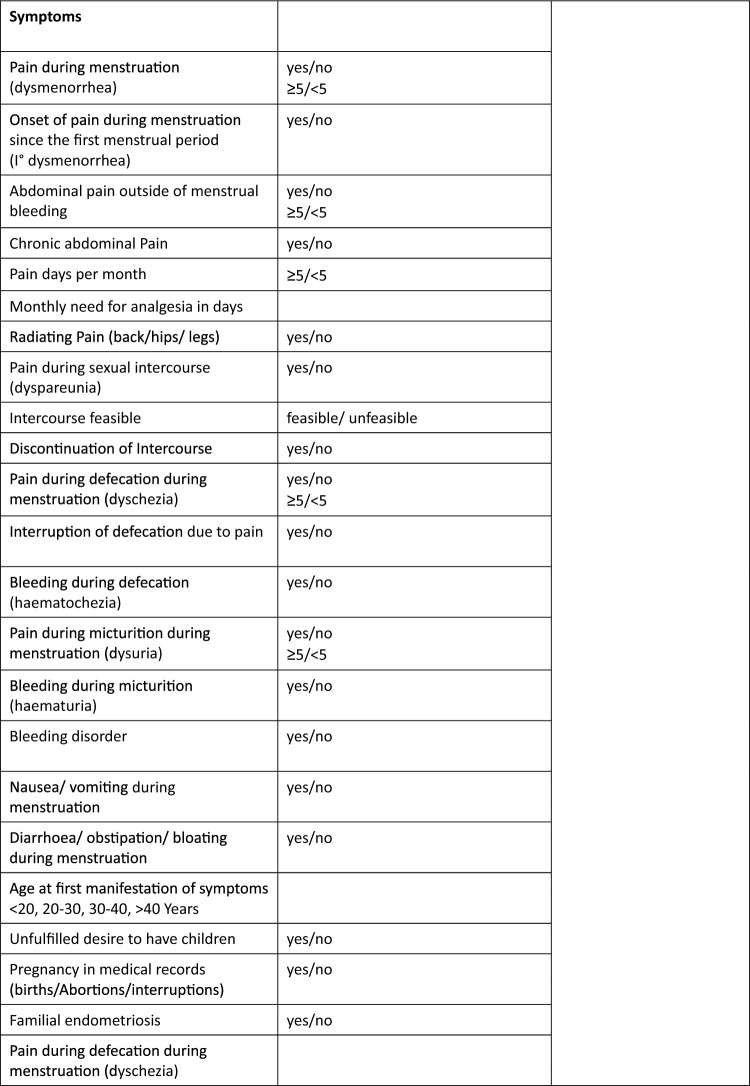
Study population selection process

This study was a retrospective analysis of preoperative questionnaires. Patients admitted to the endometriosis consultation at the academic hospital Weyertal endometriosis center of excellence are given a standardized questionnaire (Fig. 1). Patients complete the questionnaire themselves, providing a subjective assessment of their symptoms. The questionnaire must be filled out before entering the examination room. We evaluated the questionnaires from the year 2021 and included a total of 513 questionnaires after excluding duplicate entries. The patients were then divided into 2 groups: those who chose hormonal treatment and those who opted for surgery. This study only included patients who underwent surgery. The inclusion criteria were as follows: (1) a fully completed preoperative questionnaire, (2) endometriosis as the main diagnosis, (3) histological confirmation of endometriosis. A total of 233 questionnaires were included and analyzed (Fig. 2).

Intraoperative classification of endometriosis was performed according to the #ENZIAN classification. The #ENZIAN provided a useful illustration of the type and localization of endometriosis lesions. It includes the peritoneum (P), the ovary (O), adhesions involving the tubo-ovarian unit (T), and deep infiltrated endometriosis (DIE) using three compartments: A—vagina, rectovaginal space; B—uterosacral ligaments/cardinal ligaments/pelvic sidewall; and C—rectum. It also includes so-called F (other locations), which includes the adenomyosis (FA), the urinary bladder (FB), the ureters (FU), and other parts of the intestines (sigmoid colon, small bowel; FI) [9]. The numbers 1, 2, and 3 in compartments P, O, T, A, B, and C represent the extent of endometriosis. Patients were categorized into three groups based on the type and localization of their endometriosis:

- Group 1: Patients with only peritoneal endometriosis ± adnexal adhesions and adenomyosis (P ± T and FA)
- Group 2: Patients with DIE, excluding the digestive system, and/or cystic ± peritoneal and adnexal adhesions and adenomyosis (O, A, B ± P, T, and FA)
- Group 3: Patients with DIE of the digestive system (C, FI) ± other localizations.

Statistical analyses were performed using a Chi-Square calculator for a 5 × 5 (or smaller) contingency table with a significance level of < 0.05, along with descriptive statistics, confidence intervals for the mean, and One-Way ANOVA including Tukey HSD for comparing the means of three groups. The data are presented as the mean and standard deviation.

## Results

A total of 233 patients were enrolled in this study, categorized into three groups: Group 1 (n = 152), consisting of patients with P, T, and FA compartments; Group 2 (n = 57), consisting of patients with DIE without bowel involvement; and Group 3 (n = 24), consisting of patients with bowel endometriosis. There were no significant differences between the groups regarding age or BMI (Table 1).

**Table 1:** Demographic and clinical characteristics of patients in groups 1, 2, and 3

N of patients	Group 1	Group 2	Group 3	p value
	152	57	24	
Age mean ± SD	32.84 ± 7.52	35.12 ± 7.64	33,71 ± 4.46	0.1421
BMI mean ± SD kg/m^2^	24,72 ± 5.59	24,77 ± 5.68	25,78 ± 6.61	0.9903
Dysmenorrhea	148 (97.4%)	54 (94.3%)	24 (100%)	0.6382
≥ 5 VAS	140 (92.1%)	50 (87.7%)	21 (87.5%)	0.5419
< 5 VAS	8 (5.3%)	4 (7%)	3 (12.5%)	0.3976
Dyspareunia	114 (75%)	38 (66.7%)	13 (54.2%)	0.0828
≥ 5 VAS	36 (23.7%)	11 (19.3%)	6 (25%)	0.7667
< 5 VAS	78 (51.3%)	27 (47.4%)	7 (29.2%)	0.1294
Dyschezia	91 (59.8%)	38 (66.7%)	18 (75%)	0.2933
≥ 5 VAS	25 (16.4%)	12 (21.1%)	13 (54.2%)	0.0001*
< 5 VAS	66 (43.4%)	26 (45.6%)	5 (20.8%)	0.0887
Dysuria	68 (44.7%)	19 (33.3%)	10 (41.7%)	0.3298
≥ 5 VAS	8 (5.3%)	6 (10.5%)	2 (8.3%)	0.3895
< 5 VAS	60 (39.5%)	13 (22.8%)	8 (33.3%)	0.0780
Hematochezia	30 (19.7%)	7 (12.3%)	8 (33.3%)	0.0883
Hematuria	26 (17.1%)	10 (17.5%)	4 (16.7%)	0.9948
CPP	134 (88.2%)	51 (89.5%)	16 (66.7%)	0.0126*
≥ 5 VAS	91 (59.8%)	29 (50.9%)	12 (50%)	0.3971
< 5 VAS	43(28.3%)	22 (38.6%)	4 (16.7%)	0.1185
Monthly need for analgesia (days) mean ± SD	5.75 ± 4.67	5.88 ± 4.44	5.18 ± 3.41	0.7605
Nausea/vomiting	81 (53.3%)	33 (57.9%)	13(54.2%)	0.8370
Diarrhea/obstipation/bloating	140 (92.1%)	52 (91.2%)	22 (91.7%)	0.9783
AUB	105 (69.1%)	38 (66.7%)	16 (66.7%)	0.9315
Infertility	34 (22.4%)	15 (26.3%)	7 (29.2%)	0.6908

* P value with significance level 0.05

Endometriosis was classified according to the #ENZIAN, and significant differences in compartment involvement were observed across the groups (Table 2). Group 1 had a significantly higher prevalence of the P1 compartment (86.2%) compared to Groups 2 (38.6%) and 3 (45.8%) (p = 0.00001). Group 2 had a higher prevalence of the O compartment compared to Group 3, but the difference was not statistically significant (p = 0.4750). Notably, Groups 2 and 3 had a higher prevalence of the T compartment, with 57.9% of Group 2 and 58.3% of Group 3 compared to just 7.1% in Group 1 (p = 0.00001). Group 3 also showed a significant increase in involvement of the A and B compartments compared to Group 2. Severe (A3, B3) lesions were also more frequently observed in Group 3, particularly in the B compartment (p = 0.0043). The incidence of adenomyosis (FA) was notably lower in Group 3 (70.8%) compared to Groups 1 (89.5%) and 2 (85.9%) (p = 0.0433).

**Table 2:** #ENZIAN classification of patients in groups 1, 2, and 3

#ENZIAN	Group 1 (152 P)	Group 2 (57 P)	Group 3 (24 P)	p value
P (total)	152 (100%)	45 (78.9%)	17 (70.8%)	0.00001*
1	131 (86.2%)	22 (38.6%)	11 (45.8%)	0.00001*
2	16 (10.5%)	13 (22.8%)	5 (20.8%)	0.0535
3	5(3.3%)	10 (17.5%)	1 (4.2%)	0.0011*
O (total)		19 (33.3%)	10 (41.7%)	0.4750
bilateral		5 (8.8%)	4 (16.7%)	0.3018
unilateral		14 (24.6%)	6 (25%)	1.0000
1*		13 (11.4%)*	6 (12.5%)*	0.8430
2*		8 (7%)*	4 (8.3%)*	0.7702
3*		3 (2.6%)*	3 (6.25%)*	0.2654
T (Total)	11 (7.1%)	33 (57.9%)	14 (58.3%)	0.00001*
bilateral	3 (1.9%)	13 (22.8%)	5 (20.8%)	0.00001*
unilateral	8 (5.2%)	20 (35.1%)	9 (37.5%)	0.00001*
1*	12 (3.9%)*	13 (11.4%)*	4 (8.3%)*	0.0157*
2*	1 (0.3%)*	12 (10.5%)*	1 (2.1%)*	0.00001*
3*	1 (0.3%)*	21 (18.4%)*	14 (29.2%)*	0.00001*
A (total)		19 (33.3%)	19 (79.1%)	0.0001*
1		8 (14%)	2 (8.3%)	0.4762
2		11 (19.3%)	7 (29.2%)	0.3293
3			10 (41.7%)	0.00001*
B (total)		34 (59.6%)	22 (91.7%)	0.0043*
bilateral		13 (22.8%)	9 (37.5%)	0.1746
unilateral		21 (36.8%)	13 (54.2%)	0.1491
1*		23 (20.2%)*	8 (16.7%)*	0.6041
2*		22 (19.3%)*	14 (29.2%)*	0,1677
3*		1 (0.9%)*	9 (18.7%)*	0.00001*
C (total)			24 (100%)	
1			7 (29.2%)	
2			4 (16.7%)	
3			3 (12.5%)	
x			10 (41.7%)	
FA	136 (89.5%)	49 (85.9%)	17 (70.8%)	0.0433*
FB		1 (1.7%)	1 (4.2%)	0.5074
FI			3 (12.5%)	0.0237
FU			2 (8.3%)	0.0852
F diaphragm		1 (1.7%)	1 (4.2%)	0.5074
F umbilicus		2 (3.5%)	1 (4.2%)	1.0000

*The sub analysis of bilateral compartments (O, T, B) was carried out under taking into account the number of organs (double number of patients), 304, 114 and 48 in the group 1, group 2 and group 3 respectively

Dysmenorrhea was the most prevalent symptom, with 97.4% of patients in Group 1, 94.3% in Group 2, and 100% in Group 3 reporting it. The proportion of patients with a visual analog scale (VAS) ≥ 5 for dysmenorrhea did not differ significantly between groups. For dyspareunia, 75% of Group 1, 66.7% of Group 2, and 54.2% of Group 3 patients reported the symptom. A VAS score ≥ 5 for dyspareunia was observed in 23.7, 19.3, and 25% of the patients in Groups 1, 2, and 3, respectively (p = 0.7667). Dyschezia, which was more commonly reported in Group 3 (75%), followed by Group 2 (66.7%), and Group 1 (59.8%), showed a significant increase in the proportion of patients with severe dyschezia (VAS ≥ 5) in Group 3 compared to Groups 1 and 2 (p = 0.0001). Gastrointestinal symptoms, such as nausea/vomiting, were reported in similar proportions across all groups (Group 1 = 53.3%, Group 2 = 57.9%, Group 3 = 54.2%; p = 0.8370), while diarrhea, obstipation, and bloating were common in all groups (Group 1 = 92.1%, Group 2 = 91.2%, Group 3 = 91.7%; p = 0.9783). CPP was reported by 88.2% of Group 1, 89.5% of Group 2, and 66.7% of Group 3 (p = 0.0126), with a significantly lower incidence of CPP in Group 3. The mean number of days patients required analgesia per month was 5.75 ± 4.67 for Group 1, 5.88 ± 4.44 for Group 2, and 5.18 ± 3.41 for Group 3, with no significant difference (p = 0.7605). Infertility was reported in 22.4% of Group 1, 26.3% of Group 2, and 29.2% of Group 3. Group 3 had a higher proportion of patients with infertility compared to the other groups, but this difference did not reach statistical significance (p = 0.6908).

Other symptoms such as dysuria, hematochezia, and hematuria were observed in all groups, but no significant differences were found (p = 0.3298 for dysuria, p = 0.0883 for hematochezia, and p = 0.9948 for hematuria). Abnormal uterine bleeding (AUB) rate was also comparable across the groups, with no significant differences (p = 0.9315).

## Discussion

The present study aimed to investigate the relationship between the symptoms of endometriosis and the type of disease as classified by the #ENZIAN system. Our findings provide novel insights into how different types of endometriosis manifest with varying symptom profiles, particularly with respect to gastrointestinal symptoms. Endometriosis can often lead to a range of symptoms that can significantly impact a woman’s quality of life. The hallmark symptoms of endometriosis include CPP, dysmenorrhea, dyspareunia, and infertility [18]. Pelvic pain is often described as the most consistent and debilitating symptom, affecting up to 90% of women with the condition [19]. However, the intensity and presentation of pain can vary significantly between individuals, complicating the diagnostic process.

In our study all three groups displayed similar overall symptom profiles, with dysmenorrhea being the most prevalent symptom across all groups, while majority of the patients reported severe dysmenorrhea (VAS ≥ 5). CPP was another prominent symptom observed across all groups. However, Group 3 exhibited a significantly lower prevalence of CPP compared to Groups 1 and 2. This unexpected finding might be explained by the fact that DIE involving the bowel (Group 3) may not always manifest with the same intensity of pelvic pain as seen in more superficial peritoneal or ovarian endometriosis. This observation is consistent with studies suggesting that patients with bowel endometriosis may experience different pain profiles, sometimes less acute but potentially more persistent in nature, particularly when gastrointestinal symptoms dominate [20] and it is possible that the severity of pain in Group 3 patients could be masked by the pronounced dyschezia, leading to a lower reported prevalence of CPP. Our finding of a lower chronic pelvic pain (CPP) prevalence in patients with bowel endometriosis (Group 3) compared to other forms of endometriosis suggests differences in pain mechanisms, with important clinical implications. Deep bowel lesions often provoke more localized pain (e.g., dyschezia) rather than diffuse pelvic pain due to distinct visceral innervation and referred pain patterns [21]. Such lesions may also induce visceral hypersensitivity by upregulating neural pathways, leading to IBS-like gastrointestinal pain perception instead of widespread pelvic pain [22]. Clinically, women with bowel involvement may present with atypical pain distribution (primarily gastrointestinal or referred back pain) and lack classic CPP, so deep endometriosis should be considered even if CPP is absent. Consistent with our results, ENZIAN-based studies have found that lesion extent or location does not reliably predict pain severity [23]. These observations underscore the complex pathophysiology of endometriosis pain and warrant further research into how lesion location influences pain pathways. Future studies should employ pain mapping and assess central sensitization to clarify why some patients with deep lesions develop CPP while others do not, guiding more personalized pain management.

In terms of infertility, no significant differences were found across the three groups. Our findings align with previous studies that show infertility may not always correlate directly with the type or location of endometriotic lesions [4].

Vercellini et al. investigated pain syndrome, including dyspareunia, in 1000 patients. They compared 2 groups, patients with absent/mild dyspareunia and with moderate/severe dyspareunia. The data showed the significantly higher prevalence of absent/mild dyspareunia regardless of the endometriosis stage according to the AFS classification [24]. Montanari et al. identified a statistically significant association between involvement of the ENZIAN compartment B and the presence of dyspareunia [25]. In our analysis, we compared the prevalence of dyspareunia according to #ENZIAN. The rate of mild dyspareunia (VAS < 5) was higher in all three groups. There was no statistically significant difference in dyspareunia across all groups, regardless of the severity of dyspareunia and involved compartments according to #ENZIAN (Table 1). Furthermore, if comparing groups 1 and 3, dyspareunia occurred significantly higher (p = 0.0343) in patients with the P compartment. In contrast to Montanari et al., no difference was found between groups with and without B compartment (groups 1 and 2). Notably, the incidence of adenomyosis was also the same in groups 1 and 2 and significantly lower in Group 3 (Table 2). Therefore, we can make 2 conclusions. First, the role of adenomyosis in dyspareunia may be more significant than that of deep-infiltrating endometriosis. Second, we should not underestimate the role of peritoneal endometriosis in dyspareunia.

Previous studies indicated that gastrointestinal symptoms such as dyschezia and nausea are more common in patients with bowel endometriosis (#ENZIAN C and FI) [16, 24]. In contrast, in our analysis, there was no statistically significant difference in terms of dyschezia when considering it as a symptom in general and not its severity. However, considering severe dyschezia (VAS ≥ 5), the rate was significantly higher (p = 0.0001) in Group 3. The significantly higher incidence of severe dyschezia in Group 3 highlights the impact of bowel endometriosis on gastrointestinal function and quality of life.

Gastrointestinal symptoms, including diarrhea, obstipation, bloating, and nausea, were frequently reported across all groups, reflecting the widespread nature of gastrointestinal involvement in endometriosis. The prevalence of such symptoms was notably high in all three groups, without statistically significant difference across the groups (Table 1). This doesn´t align with existing literature that suggests a direct correlation between the severity of gastrointestinal symptoms and the extent of deep infiltrating endometriosis, particularly involving the bowel (C and FI compartments) [16]. There was no significant difference in hematochezia when comparing all groups (p = 0.0883) or group 3 with group 1 (p = 0.1324), but there was a significant difference when comparing group 3 with group 2 (p = 0.0259). On the one hand, this finding supports previously published data on the association between the C compartment and blood in the stool in patients with DIE. On the other hand, interestingly, we found no difference between patients with C compartments and only P compartments.

While the prevalence of dysuria was high in all groups, hematuria occurs much less. It is to be highlighted, that only 2 patients had bladder endometriosis (FB compartment). No significant differences were observed between groups for dysuria and hematuria, p = 0,3298 and 0,9948, respectively.

## Conclusion

The use of validated symptom questionnaires has been suggested to aid in the early identification of women at high risk for endometriosis [26]. This focuses on the key symptoms of endometriosis and assess their impact on a woman’s daily functioning. The thorough anamnesis of the symptoms can indicate the presence of the disease endometriosis and ensuring timely intervention. However, the extent of the disease does not always correlate with symptom severity, further highlighting the limitations of symptom-based diagnosis [6].

The present study highlights the relationship between symptoms and the type of endometriosis classified by the #ENZIAN system. All three groups displayed similar overall symptom profiles. A correlation was found only between severe dyschezia (VAS ≥ 5) and the C compartment, in the prevalence of CPP (Group 1 and Group 2 exhibited higher prevalence of CPP compared to Group 3), as well as between dyspareunia and adenomyosis (FA compartment). Therefore, additional diagnostic tools are necessary. In recent years, advances in non-invasive imaging techniques, such as transvaginal ultrasound and magnetic resonance imaging, have provided valuable adjuncts to the diagnostic process [27, 28]. While these imaging modalities can help identify DIE and endometriomas, they are less effective in detecting superficial peritoneal lesions, which can also cause significant symptoms. For this reason, even though symptom questionnaires are not definitive diagnostic tools, they may serve as an important starting point for further investigation and referral for surgical evaluation. This limitation of imaging tools underscores the importance of considering a comprehensive symptom profile in conjunction with imaging and laparoscopic findings for a more accurate diagnosis.

## Limitations and bias

While the study provides valuable insights into the symptomatology of endometriosis classified by the #ENZIAN system, there are several limitations to consider. The retrospective nature of the study and reliance on preoperative questionnaires mean that the data are subject to recall bias, and the severity of symptoms might have been underreported or overreported by patients. However, we believe that even a prospective study would likely yield similar results. This is because, despite the retrospective nature, we used prospectively collected data, as it is our routine practice to systematically document all surgical findings using the #ENZIAN classification.

## Data Availability

No datasets were generated or analysed during the current study.
